# Accumulation of Advanced Glycation End-Products and Activation of the SCAP/SREBP Lipogenetic Pathway Occur in Diet-Induced Obese Mouse Skeletal Muscle

**DOI:** 10.1371/journal.pone.0119587

**Published:** 2015-03-09

**Authors:** Raffaella Mastrocola, Massimo Collino, Debora Nigro, Fausto Chiazza, Giuseppe D’Antona, Manuela Aragno, Marco A. Minetto

**Affiliations:** 1 Department of Clinical and Biological Sciences, University of Turin, Turin, Italy; 2 Department of Drug Science and Technology, University of Turin, Turin, Italy; 3 Department of Molecular Medicine, University of Pavia, Pavia, Italy; 4 Division of Endocrinology, Diabetology and Metabolism, Department of Medical Sciences, University of Turin, Turin, Italy; INRA, FRANCE

## Abstract

Aim of this study was to investigate whether advanced glycation end-products (AGEs) accumulate in skeletal myofibers of two different animal models of diabesity and whether this accumulation could be associated to myosteatosis. Male C57Bl/6j mice and leptin-deficient ob/ob mice were divided into three groups and underwent 15 weeks of dietary manipulation: standard diet-fed C57 group (C57, n = 10), high-fat high-sugar diet-fed C57 group (HFHS, n = 10), and standard diet-fed ob/ob group (OB/OB, n = 8). HFHS mice and OB/OB mice developed glycometabolic abnormalities in association with decreased mass of the gastrocnemius muscle, fast-to-slow transition of muscle fibers, and lipid accumulation (that occurred preferentially in slow compared to fast fibers). Moreover, we found in muscle fibers of HFHS and OB/OB mice accumulation of AGEs that was preferential for the lipid-accumulating cells, increased expression of the lipogenic pathway SCAP/SREBP, and co-localisation between AGEs and SCAP-(hyper)expressing cells (suggestive for SCAP glycosylation). The increased expression of the SCAP/SREBP lipogenic pathway in muscle fibers is a possible mechanism underlying lipid accumulation and linking myosteatosis to muscle fiber atrophy and fast-to-slow transition that occur in response to diabesity.

## Introduction

Obesity-associated diabetes (“diabesity”) is a serious health problem with escalating global prevalence that follows the escalating global epidemic of overweight and obesity (“globesity”) [1]. In both human patients and animal models, it is characterized by severe insulin resistance and hyperglycemia which have a major role in the pathogenesis of complications affecting different organs. In fact, hyperglycemia is accompanied by an accelerated rate of formation of advanced glycation end-products (AGEs), such as carboxy-methyllysine (CML) and carboxy-ethyllysine (CEL), which are reactive compounds formed by the nonenzymatic reaction between glucose-derived intermediates and free amino groups of proteins. It has been previously demonstrated that the accumulation of AGEs in tissues promotes diabetic nephropathy [2,3], vascular dysfunction [4–6], diabetic cardiomyopathy [7], impaired bone formation and increased risk of osteoporosis [8,9], cancer cell proliferation [10]. Moreover, AGEs accumulation in liver promotes hepatosteatosis and dyslipidemia [11–13] through the activation of SCAP (sterol-regulatory element binding protein cleavage-activating protein)—SREBP (sterol-regulatory element binding protein) lipogenetic pathway [14].

Besides their role in the pathogenesis of diabetic complications, recent data also showed that AGEs may impair insulin action through different mechanisms: they interfere with the complex molecular pathway of insulin signaling, modify the insulin molecule, and decrease insulin secretion [15–19]. As the skeletal muscle cells account for most of the insulin-stimulated glucose uptake, it may be hypothesized that AGEs accumulate in skeletal muscle in response to diabesity and contribute to both insulin resistance and occurrence of muscle degenerative changes known as “fatty atrophy” (i.e., the combination of muscle fiber atrophy, fast-to-slow transition of muscle fibers, and myosteatosis) [20]. Consistently, accumulation of AGEs in skeletal muscles of elderly rats has recently been documented by Ramamurthy et al. [21], who suggested that the myofibrillar protein glycosylation is a possible mechanism underlying the age-related related decline in muscle function. Moreover, it has been observed that elderly rats experimentally exposed to rotator cuff tear developed fatty atrophy in association with a marked accumulation of AGEs and a significant accretion of macrophages in areas of fat accumulation [22]. Fatty atrophy and fast-to-slow transition of muscle fibers have also been documented to occur, in association with glycometabolic abnormalities, in diet-induced obesity animal models [23–27]. In other words, the diet-induced muscle degenerative changes resemble the aged-related changes which have been attributed, at least partially, to AGEs accumulation in muscle fibers.

To our knowledge, no previous study has documented the accumulation of AGEs in skeletal muscles in response to diet-induced obesity. Therefore, the aim of this study was to investigate whether AGEs accumulate in skeletal myofibers of two different animal models of diabesity and whether this accumulation could be associated to activation of the SCAP/SREBP lipogenetic pathway that may, in turn, trigger lipid production and accumulation.

## Materials and Methods

### Animals and treatments

Male C57Bl/6j mice (Charles River Laboratories, Calco, Italy) and leptin-deficient ob/ob mice (Jackson Laboratories, Bar Harbor, ME, USA) aged 4 weeks were cared for in compliance with the European Council directives (No. 86/609/EEC) and with the Principles of Laboratory Animal Care (NIH No. 85–23, revised 1985). The scientific project was approved by the Ethical Committee of the Turin University (permit number: D.M. 94/2012-B). The animals were divided into three groups: standard diet-fed C57 group (C57, n = 10), high-fat high-sugar diet-fed C57 group (HFHS, n = 10), and standard diet-fed ob/ob group (OB/OB, n = 8). The duration of interventions (standard diet and HFHS diet) was 15 weeks. Standard diet composition was: 70% of calories in carbohydrates (55% from corn starch and 15% from maltodextrin), 10% of calories in fat (5% from soybean, 5% from butter). High-fat high-sugar diet composition was: 45% of calories in carbohydrates (15% from corn starch and 30% from sucrose), 40% of calories in fat (2% from soybean, 38% from butter). All groups received drink and food ad libitum.

### Procedures and analyses

Body weight and food intake were recorded weekly. Fasting glycemia was measured at the start of the protocol and every 4 weeks by saphenous vein puncture using a glucometer (GlucoGmeter, Menarini Diagnostics, Florence, Italy). After 15 weeks, the oral glucose tolerance test (OGTT) was performed on 6-hours fasted mice. A glucose solution was administered orally at 2 g/kg body weight and plasma glucose levels were measured at several time points for 2 hours after glucose loading. Mice were then anesthetized and killed by cardiac exsanguination. Blood was collected and the gastrocnemii were rapidly removed. Plasma lipid profile was determined by standard enzymatic procedures using reagent kits (Hospitex Diagnostics, Florence, Italy). Plasma insulin level was measured using an enzyme-linked immunosorbent assay (ELISA) kit (Mercodia AB, Uppsala, Sweden). The right gastrocnemius was cryoprotected in OCT (Optimal Cutting Temperature) compound (VWR, Milano, Italy) and frozen in N_2_ for cryostatic preparations. The left gastrocnemius was frozen in N_2_ and stored at −80°C for protein analysis.

### Gastrocnemius lipid content

For tissue triglyceride content determination, colorimetric assay kit was used after lipid extraction (Triglyceride Quantification Kit, Abnova Corporation, Aachen, Germany).

Gastrocnemius intramyocellular lipid (IMCL) accumulation was evaluated by Oil Red O staining on 10 μm cryostatic sections. Stained tissues were viewed under an Olympus Bx4I microscope (40x magnification) with an AxioCamMR5 photographic attachment (Zeiss, Göttingen, Germany).

### Preparation of tissue extracts

Gastrocnemius total proteins were extracted as previously described [14]. Total extracts were obtained from 10% (w/v) left gastrocnemius homogenates in RIPA buffer (0.5% Nonidet P-40, 0.5% sodium deoxycholate, 0.1% SDS, 10 mmol/l EDTA, and protease inhibitors). After 40 minutes of incubation in ice, samples were sonicated and cleared by centrifugation at 15,000 g at 4°C for 40 min. Supernatants were removed and protein content was determined using the Bradford assay. Protein extracts were stored at −80°C until use.

### Western blotting

Equal amounts of proteins were separated by SDS-PAGE and electrotransferred to nitrocellulose membrane (GE-Healthcare Europe, Milano, Italy). Membranes were probed with rabbit anti-SREBP-1c (Abcam, Cambridge, UK), rabbit anti-SCAP (Abcam), rabbit anti-ACC and anti-pACC^Ser79^ (Cell Signaling Technology, Danver, MA, USA), mouse anti-FASN (Santa Cruz Biotechnology, CA, USA), mouse anti-MHC I (Abcam), rabbit anti-MHC IIA (Novus Biologicals, Cambridge, UK), rabbit anti-MHC IIB (Proteintech, Chicago, IL, USA), rabbit anti-MHC (Abcam), mouse anti-CML (R&D System, Minneapolis, MN, USA), mouse anti-CEL (TransGenic, Kobe, Japan), mouse anti-RAGE (Abcam), goat anti-AGE-R1 (Santa Cruz Biotechnology) followed by incubation with appropriated HRP-conjugated secondary antibodies (BioRad, Hercules, CA, USA).

Proteins were detected with Clarity Western ECL substrate (BioRad) and quantified by densitometry using analytic software (Quantity-One, Bio-Rad). Results were normalized with respect to alpha-tubulin densitometric value.

### Muscle composition analysis

Gastrocnemius fibers were stained on serial 10 μm cryostatic sections by immunohistochemistry. Endogenous peroxidases were inactivated by incubating sections for 5 min with 0.3% H_2_O_2_. Sections were then blocked for 1 h with 3% BSA in PBS added with unconjugated goat anti-mouse IgG to prevent mouse-on-mouse interferences. Thus, sections were incubated overnight with mouse anti-MHC I and rabbit anti-MHC IIA primary antibodies followed by HRP-conjugated secondary antibodies. Sections were digitised with a high resolution camera (Zeiss) at 20x magnification. Fiber type percentages were measured on an average of 200–500 fibers per animal.

### Immunofluorescence analysis

Localization of the two AGEs CML and CEL and of the receptor for AGEs (RAGE) was assessed by indirect immunofluorescence on 10 μm gastrocnemius cryostatic sections. After blocking, sections were incubated overnight with primary antibodies and for 1 h with Cy3-labelled secondary antibodies (Dako, Glostrup, Denmark). Negative controls were prepared incubating sections only with secondary antibodies. Nuclei were stained with Hoechst dye and sections were then examined using a Leica Olympus epifluorescence microscope (Olympus Bx4I) at 10x/40x magnification and digitised with a high resolution camera (Zeiss).

### Colocalization analysis

Colocalization analysis was performed between IMCL and MHC isoforms, CML, and CEL on gastrocnemius cryostatic sections from HFHS and OB/OB mice. Briefly, after immunofluorescence, lipids were stained in the same section with the green fluorescent dye boron-dipyrromethene (BODIPY) (493/503, Molecular Probes, Carlsbad, CA, USA) for 30 min, as previously described [28]. Further, a double immunofluorescence analysis was performed for colocalization between CML and SCAP.

### Materials

All compounds were purchased from Sigma Chemical Co. (St. Louis, MO, USA), unless otherwise stated.

### Statistical analysis

The Shapiro-Wilk test was used to assess the normality of the variable distributions. One-way ANOVA followed by Bonferroni’s post-hoc test were adopted for comparisons among the three groups of animals. Data were expressed as mean ± standard deviation (SD). Threshold for statistical significance was set to P<0.05. Statistical tests were performed with GraphPad Prism 6.0 software package (GraphPad Software, San Diego, CA, USA).

## Results

### General parameters

After 15 weeks of diet, HFHS and OB/OB mice showed higher values of total body weight and epididymal fat weight and lower values of gastrocnemius weight in comparison to standard diet-fed C57 mice. A statistically significant difference in weight was also observed between OB/OB mice and HFHS mice (Table 1).

**Table 1:** General parameters obtained in standard diet fed-C57Bl/6j mice (C57), high-fat high-sucrose fed-C57Bl/6j mice (HFHS), and standard diet-fed leptin-deficient mice (OB/OB) after 15 weeks of dietary regimens.

	C57 (n = 10)	HFHS (n = 10)	OB/OB (n = 8)
Body weight (g)	27.3 ± 0.4	28.5 ± 0.6*	59.3 ± 3.8*** ^#^
Epididymal fat weight (% body weight)	2.13 ± 0.27	2.73 ± 0.24*	5.05 ± 0.61*** ^###^
Gastrocnemius weight (% heart weight)	2.64 ± 0.15	2.41 ± 0.11*	1.31 ± 0.27*** ^#^
Daily caloric intake (Kcal/die)	11.2 ± 0.6	13.6 ± 0.5***	13.9 ± 0.8***
Fasting glycemia (mg/dl)	85 ± 9	110 ± 8*	210 ± 37*** ^###^
Plasma insulin (μg/l)	0.15 ± 0.03	0.50 ± 0.17**	1.19 ± 0.15*** ^###^
Plasma triglycerides (mg/dl)	0.72 ± 0.07	0.88 ± 0.02*	1.34 ± 0.25*** ^###^
Plasma cholesterol (mg/dl)	2.47 ± 0.38	3.25 ± 0.13*	5.98 ± 2.00** ^###^

Data are means ± S.D.

*P<0.05,

**P<0.01,

***P<0.005 vs C57;

^#^ *P*<0.05,

^##^ *P*<0.001,

^###^ *P*<0.005 vs HFHS.

**Fig 1:**
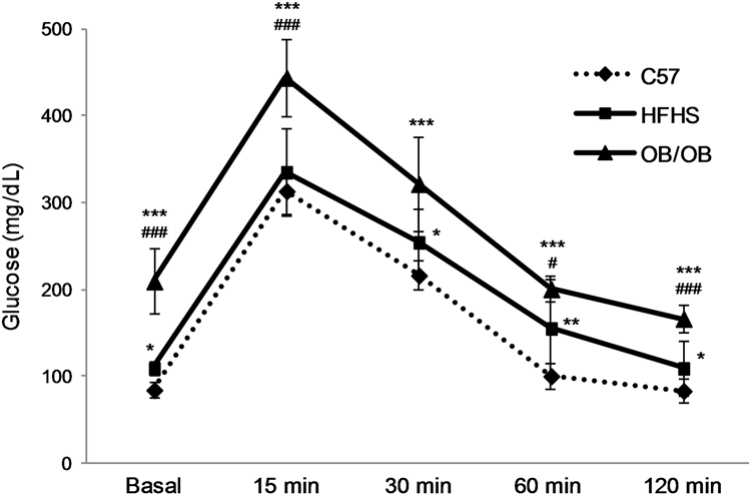
Oral glucose tolerance test. OGTT was performed on 6-hours fasted standard diet fed-C57 mice (C57, *n* = 10), high-fat high-sucrose fed-C57 mice (HFHS, *n* = 10), and standard diet-fed leptin-deficient mice (OB/OB, *n* = 8). Glycemic values before (Basal) and 15, 30, 60, 120 minutes after glucose loading are reported. Results are means ± SD. *P<0.05, **P<0.01, ***P<0.005 vs C57 ^#^ *P*<0.05, ^###^ *P*<0.005 vs HFHS.

Both HFHS and OB/OB mice had higher daily caloric intake, associated with a significant increase in plasma fasting glucose, insulin, triglyceride, and cholesterol levels in comparison to C57 mice, and OGTT revealed the onset of glucose intolerance. Statistically significant differences in glycometabolic parameters were also observed between OB/OB mice and HFHS mice (Table 1 and Fig. 1).

### Myosteatosis and increased expression of the SCAP/SREBP pathway

**Fig 2:**
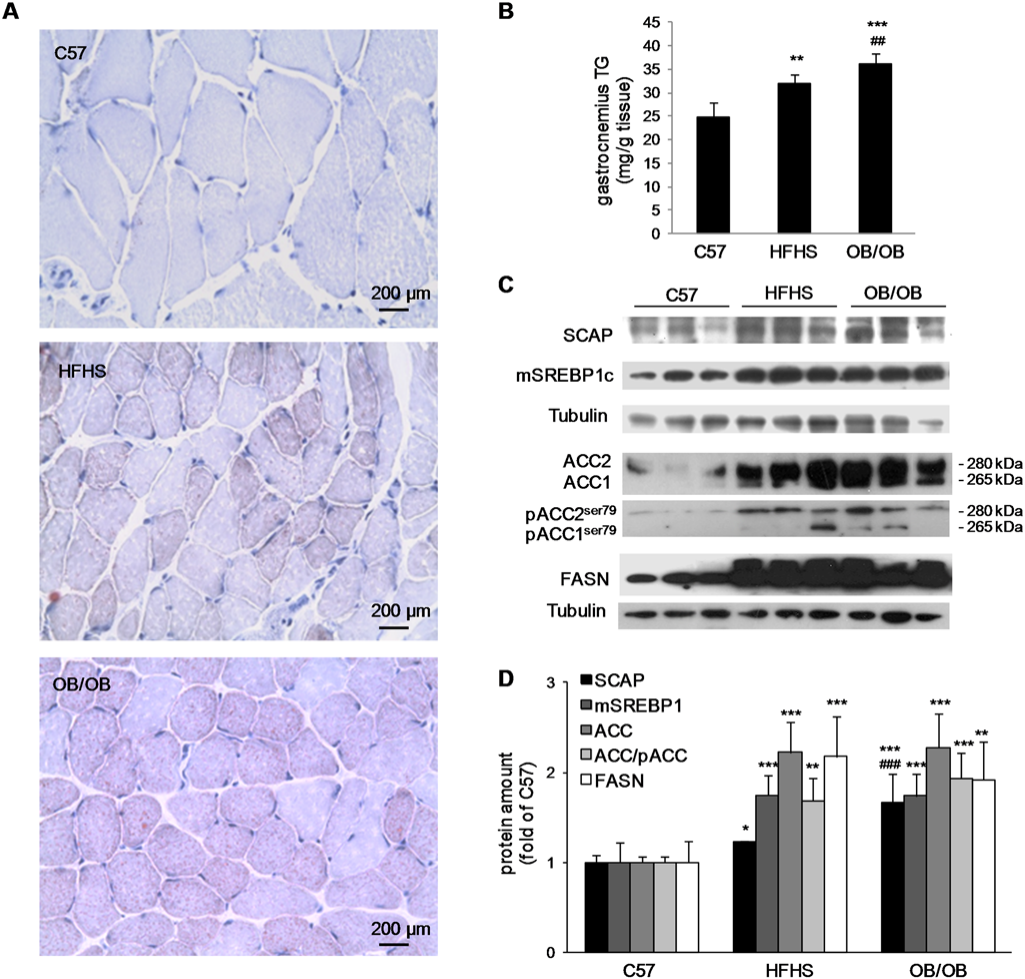
Gastrocnemius intramyocellular lipid accumulation, triglyceride content, and SCAP/SREBP pathway analysis. (A) Representative 20x magnification photomicrographs of Oil Red staining on gastrocnemius sections from C57, HFHS, and OB/OB mice performed on 8–10 mice per group. (B) Triglyceride (TG) content in gastrocnemius homogenates of the three groups. (C) Representative western blotting analysis for SCAP, mSREBP1c, ACC, pACC, and FASN. (D) Histograms report densitometric analyses of 8–10 mice per group (results were normalized with respect to alpha-tubulin densitometric value). *P<0.05, **P<0.01, ***P<0.005 vs C57; ^##^ *P*<0.01, ^###^ *P*<0.005 vs HFHS.

Oil Red O staining on gastrocnemius sections highlighted IMCL deposition in myofibers of HFHS mice, and to a greater extent, in myofibers of OB/OB mice (Fig. 2A). Accordingly, gastrocnemius triglyceride levels were higher in HFHS mice compared to C57 mice and were higher in OB/OB mice compared to both HFHS and C57 mice (Fig. 2B).

To investigate the source of triglyceride accumulation, we assessed the activation of the lipogenic pathway regulated by SREBP-1c and by its activating protein SCAP. Western blotting analysis on muscle extracts showed up-regulations of SCAP, of the active form of SREBP-1c (mSREBP-1c), and of the SREBP-1c target genes Acetyl-CoA carboxylase (ACC) and fatty acid synthase (FASN) in HFHS and OB/OB mice compared to C57 mice. Interestingly, expression of SCAP was also significantly higher in OB/OB compared to HFHS mice, while expressions of mSREBP-1c, ACC isoforms (ACC1 and ACC2), and FASN were comparable between HFHS and OB/OB mice (Fig. 2C and D). As the ACC phosphorylation leads to its inactivation, we also assessed the expression of the phosphorylated isoforms (pACC1^ser79^ and pACC2^ser79^). We found that pACC expression was lower than ACC expression in HFHS and OB/OB mice compared to C57 mice, suggesting an overall increase in the enzymatic activity.

### Muscle composition changes in relation to lipid accumulation

**Fig 3:**
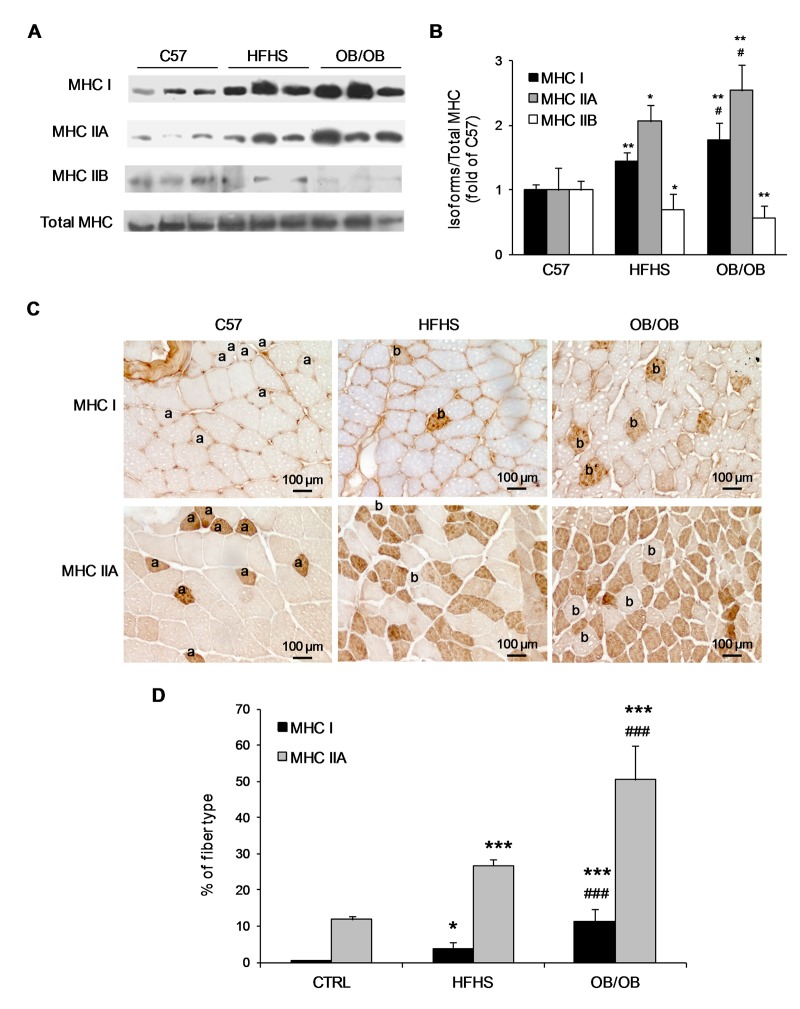
Gastrocnemius myosin heavy chain isoform composition. (A) Representative western blotting analysis for the expression of MHC I, MHC IIA, MHC IIB isoforms and total MHC on gastrocnemius total protein extracts from C57, HFHS, and OB/OB mice. (B) Histograms report densitometric analyses of MHC isoforms normalized for the total MHC content of 8–10 mice per group. (C) Representative 20x magnification photomicrographs of immunohistochemistry analysis for MHC I and MHC IIA on gastrocnemius frozen sections of the three groups. MHC I positive myofibers are marked with *a* in serial sections; MHC IIA positive myofibers are marked with *b* in serial sections. (D) Histograms report the percentage of MHC I and MHC IIA positive fibers counted on 200–500 fibers per animal (8–10 mice per group). *P<0.05, **P<0.01, ***P<0.005 vs C57; ^#^ *P*<0.05, ^###^ *P*<0.005 vs HFHS.

A higher expression of MHC I and MHC IIA and a lower expression of MHC IIB were observed by western blotting analysis in HFHS and OB/OB mice compared to C57 mice. Interestingly, the expression of MHC I and MHC IIA was also significantly higher in OB/OB mice compared to HFHS mice (Fig. 3A and B).

Consistently, immunohistochemical analyses on gastrocnemius sections of HFHS and OB/OB mice showed an increased number of MHC I and type IIA fibers compared to C57 mice (Fig. 3C). Specifically, a 5-fold and a 10-fold increase in MHC I fibers, and a 2.5-fold and a 5-fold increase in MHC IIA fibers were observed in HFHS and OB/OB mice, respectively, compared to C57 mice. Interestingly, the percentages of MHC I and MHC IIA fibers were also significantly higher in OB/OB mice compared to HFHS mice (Fig. 3D).

**Fig 4:**
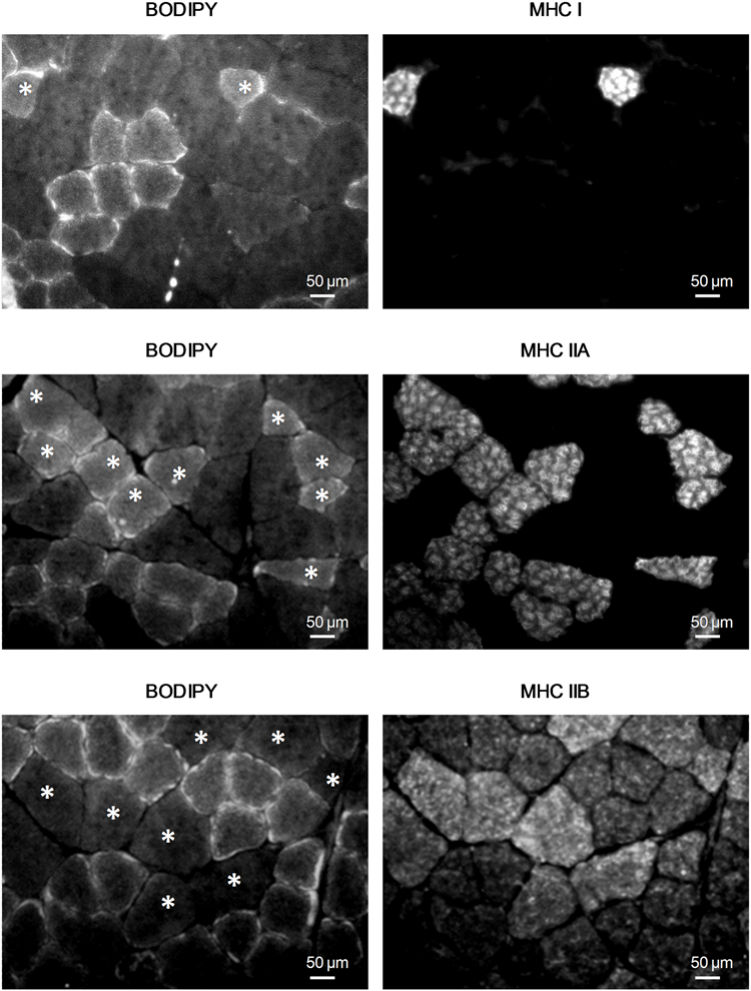
Gastrocnemius colocalization of IMCL and oxidative MHC isoforms. The fluorescent stain BODIPY was used to visualize IMCL accumulation in association with immunofluorescence analysis for MHC I, MHC IIA and MHC IIB on gastrocnemius sections from HFHS and OB/OB mice (5 mice per group). Representative 32x magnification photomicrographs of an HFHS mouse showing IMCL overlapping only with MHC I and IIA isoforms. Double fluorescent staining was performed on the same section and the MHC isoform positive myofibers shown on the right image are starred on the left image.

The use of BODIPY, a fluorescent staining to visualize IMCL, in association with immunofluorescence analysis on gastrocnemius sections of HFHS mice, showed that IMCL accumulated specifically in MHC I and MHC IIA fibers, while MHC IIB fibers did not accumulate lipids at all (Fig. 4). Similar results were obtained in OB/OB mice (data not shown).

### AGEs accumulation and AGEs receptors expression

**Fig 5:**
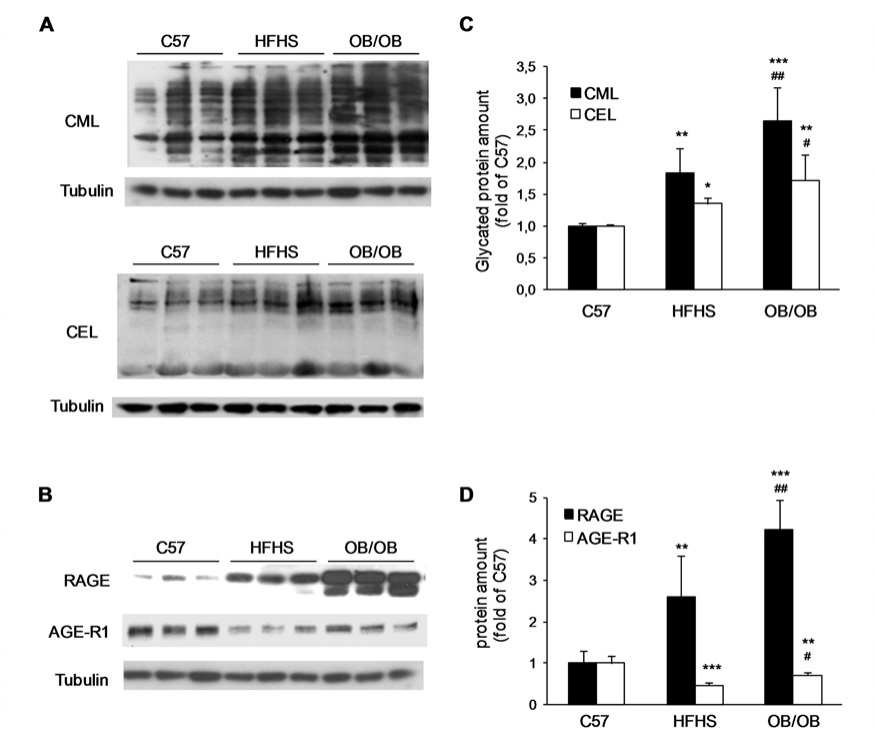
Gastrocnemius levels of glycated proteins and AGEs receptors expression. Representative western blotting analysis for CML and CEL modified-proteins (A) and for RAGE and AGE-R1 expression (B) performed on gastrocnemius total protein extracts from C57, HFHS, and OB/OB mice. (C,D) Histograms report densitometric analyses of 8–10 mice per group. *P<0.05, ***P<0.005 vs C57; ^#^ *P*<0.05, ^##^ *P*<0.01 vs HFHS.

Western blotting analysis on gastrocnemius protein extracts showed increased levels of CML- and CEL-modified proteins (Fig. 5A and C), increased expression of RAGE, and decreased expression of the AGEs receptor-1 (AGE-R1) (Fig. 5B and D) in HFHS and OB/OB mice compared to C57 mice. Interestingly, the levels of CML- and CEL-modified proteins and the expression of RAGE were also significantly higher in OB/OB mice compared to HFHS mice.

**Fig 6:**
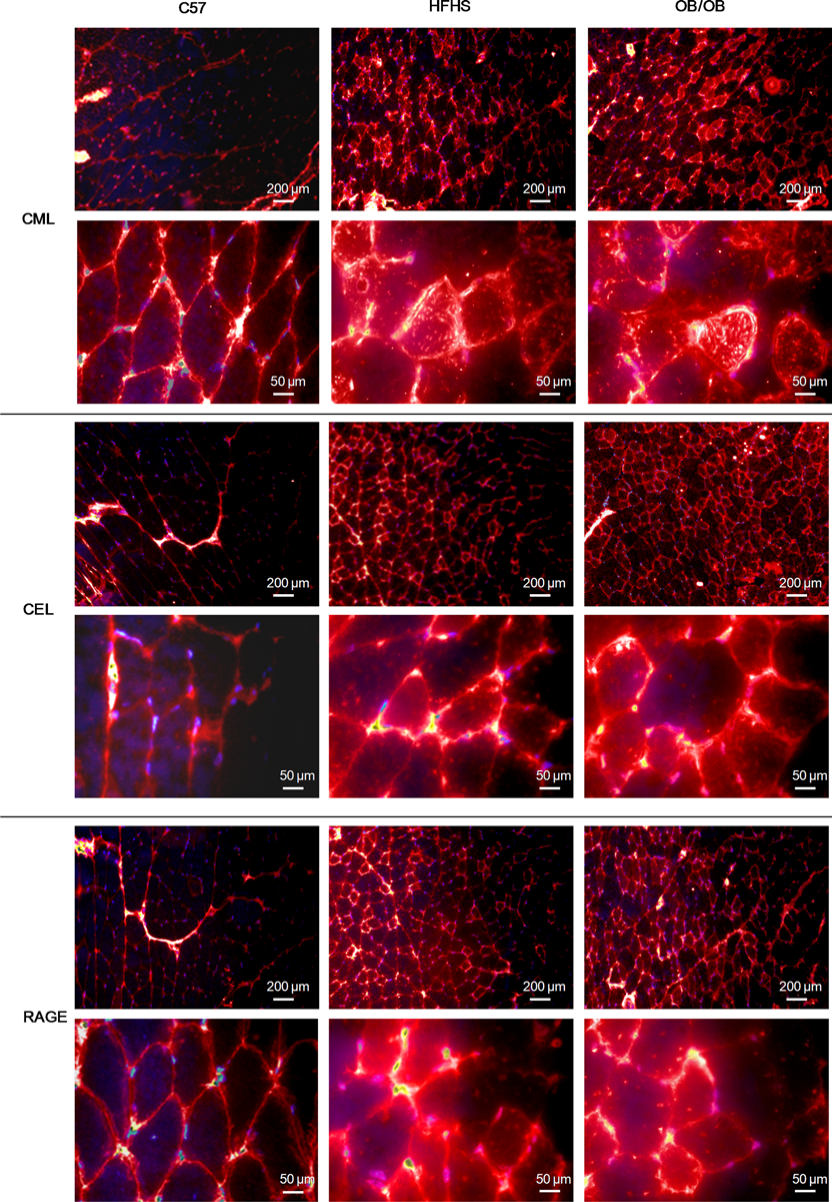
Gastrocnemius localization of AGEs. Representative 10x/40x magnification photomicrographs of immunofluorescence analyses for CML, CEL, and RAGE on gastrocnemius sections from C57, HFHS, and OB/OB mice performed on 8–10 mice per group.

The accumulation of CML and CEL, and the increased expression of RAGE in HFHS and OB/OB mice compared to C57 were confirmed by immunofluorescence analysis on gastrocnemius sections (Fig. 6). In particular, a prevalent cytosolic accumulation for CML and an extracellular localization for CEL were observed, while RAGE was expressed along the membranes of muscle fibers.

### Relation between lipid and AGEs accumulation

**Fig 7:**
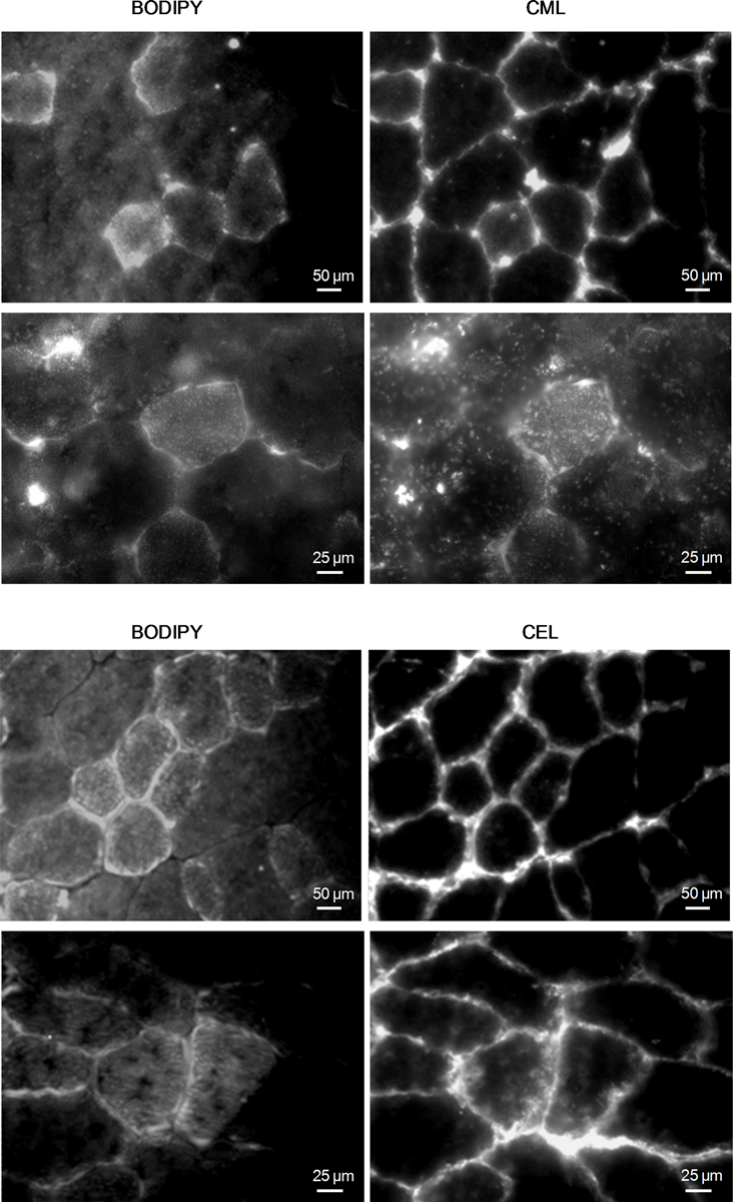
Gastrocnemius colocalization of IMCL and AGEs. BODIPY stain was used to visualize IMCL in association with immunofluorescence analysis for CML and CEL on gastrocnemius sections from HFHS and OB/OB mice (5 mice per group). Representative 32x/63x magnification photomicrographs of an HFHS mouse show wide overlapping between IMCL positive myofibers (left panels) and CML- or CEL-positive myofibers (right panels).

**Fig 8:**
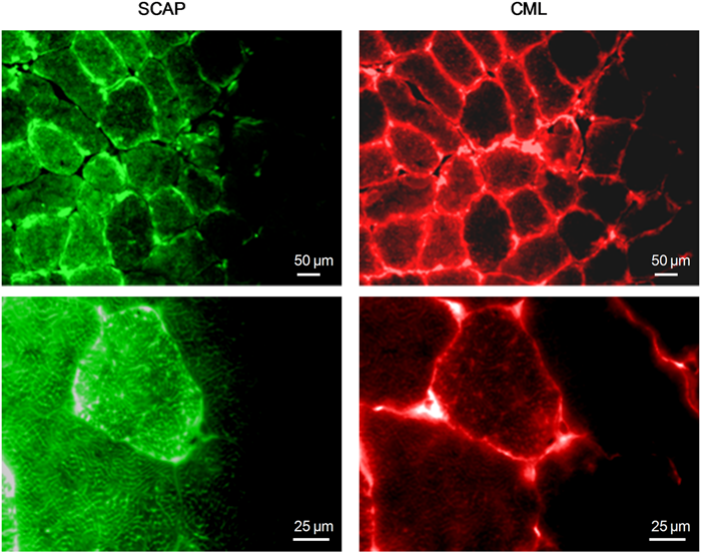
Gastrocnemius colocalization between SCAP and CML. Double immunfluorescent analysis for CML and SCAP was performed on gastrocnemius sections from HFHS and OB/OB mice (5 mice per group). Representative 32x/100x photomicrographs of an HFHS mouse show wide overlapping between CML and SCAP in the cytosolic compartment of myofibers.

To investigate the relationship between AGEs and IMCL accumulation, we performed a double fluorescence analysis on gastrocnemius sections of HFHS and OB/OB mice. IMCL staining was co-localized in the same myofibers with CML and CEL (Fig. 7). Specifically, CML widely overlapped with SCAP within the myofibers, suggesting a considerable glycosylation of SCAP (Fig. 8). Similar results were obtained in OB/OB mice (data not shown).

## Discussion

In the present study, we observed that C57 mice fed with HFHS diet and OB/OB mice fed with standard diet for 15 weeks developed glycometabolic abnormalities in association with decreased mass of the gastrocnemius muscle, fast-to-slow transition of muscle fibers, and lipid accumulation that occurred preferentially in slow compared to fast fibers. Moreover, this study is the first showing in muscle fibers of HFHS and OB/OB mice accumulation of AGEs that was preferential for the lipid-accumulating cells, increased expression of the lipogenic pathway SCAP/SREBP, and co-localisation between AGEs and SCAP-(hyper)expressing cells suggestive for SCAP glycosylation.

### AGEs accumulation and increased expression of the SCAP/SREBP pathway in response to diabesity

SREBPs are transcription factors that belong to the basic helix-loop-helix leucine zipper family of DNA binding proteins and activate the synthesis of cholesterol, fatty acids, and triglycerides in all organs [29,30]. The SREBP family includes three isoforms with overlapping functions. SREBP-1a activates cholesterol and fatty acid synthesis, SREBP-1c primarily activates fatty acid synthesis, SREBP-2 activates cholesterol synthesis [29,30]. It has been previously demonstrated that hyperactivation of SREBP-1c in liver causes triglyceride accumulation which may lead to hepatosteatosis [11–13] and that the primary modulators of SREBP-1c are insulin [31–34], insulin-like growth factor-1 [34], inflammatory cytokines such as tumor necrosis factor-alpha [35], while its primary activator is SCAP which escorts SREBP-1c from the endoplasmic reticulum to the Golgi, where it is cleaved by two proteases. Thereafter, the cleaved N-terminal fragment (nSREBP-1c) enters the nucleus, binds to the sterol-regulatory elements, and increase gene transcription [3,13]. Yuan et al. [3] recently showed that CML increased lipid synthesis in human mesangial cells via increasing SCAP expression and intracellular translocation from the endoplasmic reticulum to the Golgi. Consistent with this finding, high levels of CML and activation of the SCAP/SREBP pathway were observed in liver of fructose-drinking mice [14].

In the present study, we observed an association among diet-induced glycometabolic abnormalities, accumulation of CML- and CEL-modified proteins, increased expression of SCAP, and lipid accumulation in mouse gastrocnemius. Overall, this association suggests that: i) AGEs accumulation (i.e., SCAP glycosylation) and hyperinsulinemia combine to cause SREBP-1c activation in muscle fibers; ii) increased expression of the SCAP/SREBP pathway in muscle fibers is a possible mechanism underlying lipid accumulation and development of myosteatosis. A limitation of our study is that it is correlative in nature: therefore, it is not possible either to demonstrate a clear causal link between AGEs accumulation and SREBP-1c activation or to identify which of the two factors (SCAP glycosylation vs hyperinsulinemia) has a greater effect on SREBP-1c expression. Moreover, we cannot rule out that AGEs accumulation may affect other transcriptional factors involved in fatty acid synthesis and lipid accumulation. For instance, a very recent study has demonstrated in colorectal cancer cells and hepatoblastoma cells a role of AGEs in the modulation of the activity of the carbohydrate response element binding protein (ChREBP) [36], which is another transcriptional regulator of de novo fatty acid synthesis, highly expressed in adipose and muscle tissues [37,38].

Another original result of this study was that the greater the diet-induced glycometabolic abnormalities, the higher the RAGE expression in mouse gastrocnemius. Moreover, AGE-R1 expression was lower in HFHS and OB/OB mice compared to C57 mice. AGE-R1 is responsible for detoxification and clearance of AGEs [39], while RAGE is a multiligand receptor belonging to the immunoglobulin superfamily of cell surface molecules acting as a counter-receptor for diverse molecules [4,39,41]. In fact, not only AGEs, a number of other ligands that tend to be released or accumulated in tissues during inflammatory disorders, immune responses, and chronic degenerative diseases also interact with RAGE [4,39–41]. RAGE engagement with its ligands activates multiple cellular signaling pathways (such as MAP kinases, SAPK/JNK, rho-GTPases, JAK/STAT and nuclear factor-kB) that are associated with increased production of pro-inflammatory mediators and further enhances the expression of RAGE [39,40]. Therefore, ligands and RAGE interaction forms a positive feedback loop that converts acute inflammatory stimulus into sustained cellular dysfunction and further magnifies insulin resistance and tissue damage. Modulation of RAGE signaling pathway could represent a promising target to counteract diabesity-related insulin resistance and fatty atrophy.

### Muscle fiber atrophy and fast-to-slow transition in response to diabesity

Mammalian skeletal muscle comprises different fiber types, whose identity is first established during embryonic development by intrinsic myogenic control mechanisms and is later modulated by neural and hormonal factors [42,43]. The differential distribution of MHC isoforms defines three major fiber types containing a single isoform: slow oxidative fibers (containing MHC I), fast oxidative-glycolytic fibers (MHC IIA), and fast glycolytic fibers (MHC IIB) [44]. It has been known for a long time that muscle fibers can change their properties through a “quantitative mechanism” (i.e., going through atrophy or hypertrophy), and that such mechanism is related to a fiber type-specific regulation of MHC gene expression. In other words, the adaptive plasticity of muscle fibers is mainly, although not exclusively, related to the pattern of MHC isoform expression [45].

Previous animal studies have documented that a fast-to-slow (glycolytic-to-oxidative) phenotype transition occurs in response to diet-induced obesity and represents an early adaptation to counteract the lipotoxicity-related impairment of muscle oxidative capacity through the enhancement of the muscle oxidative potential and/or mitochondrial content [23–25,27]. In the present study, we confirmed and extended these previous findings: in fact, we observed that the muscle fiber atrophy (indirectly inferred from the decreased mass of the gastrocnemius muscle) and the fast-to-slow phenotype transition were associated with accumulation of AGEs and increased expression of the SCAP/SREBP pathway that were preferential for the lipid-accumulating slow muscle fibers. Although association does not imply causality, we suggest that SREBP-1c up-regulation may represent a molecular mechanism linking myosteatosis to muscle fiber atrophy and fast-to-slow transition that occur in response to diabesity. Consistently, recent investigations have shown that SREBP-1a and -1c not only regulate cholesterol and fatty acid metabolism, but are also involved in the regulation of muscle mass and muscle cell differentiation [46,47]. In fact, their overexpression in the tibialis anterior muscle of mice promoted tibialis anterior weight reduction (in keeping with our observation of gastrocnemius weight reduction) and decrease in fiber cross sectional area [47] that were attributed to a marked decrease in the expression of myogenic regulatory factors and sarcomeric proteins (i.e., decrease in protein synthesis) not compensated for by a small decrease in proteolysis [47,48]. Moreover, SREBP-1c has been demonstrated to up-regulate the expression of myostatin [49], a well-known negative regulator of muscle mass which inhibits muscle cell proliferation and protein synthesis [50]. Overall, the atrophic effect of SREBP-1 overexpression may represent a negative feedback loop to control muscle hypertrophy [47] that could be triggered, in the present experimental conditions as well as in pathological states, by increased levels of insulin. On the other hand, the fast-to-slow isoform transition could represent a positive feed-forward mechanism to enhance the oxidative capacity of muscle fibers.

## Conclusions

We observed in the gastrocnemius muscle of diet-induced obese mice accumulation of AGEs and increased expression of the SCAP/SREBP pathway in association with glycometabolic abnormalities, decreased muscle mass, fast-to-low transition of muscle fibers, and myosteatosis. Future studies will be required to demonstrate a clear causal link between AGEs accumulation and SREBP activation and to prove the role of SREBPs as integrators of signals coming from nutrition, cytokines, and anabolic hormones (i.e., insulin) toward a control of muscle mass and composition and their changes induced by diabesity.

## Supporting Information

S1 Abbreviation List (DOCX)
