# Supplementary material for: Accumulation of Advanced Glycation End-Products and Activation of the SCAP/SREBP Lipogenetic Pathway Occur in Diet-Induced Obese Mouse Skeletal Muscle
Source: PLoS One. 2015 Mar 9;10(3):e0119587. doi: 10.1371/journal.pone.0119587 (PMC4353621; doi:10.1371/journal.pone.0119587)
Supplement: S1 Abbreviation List — (DOCX) [file pone.0119587.s001.docx]

ACC: Acetyl-CoA carboxylase

AGEs: advanced glycation end-products

CEL: carboxy-ethyllysine

ChREBP: carbohydrate response element binding protein

CML: carboxy-methyllysine

FASN: fatty acid synthase

HFHS: high-fat high-sugar

IMCL: intramyocellular lipid

MHC: myosin heavy chain

OGTT: oral glucose tolerance test

RAGE: receptor for advanced glycation end-products

SCAP: sterol-regulatory element binding protein cleavage-activating protein

SREBP: sterol-regulatory element binding protein

TG: triglyceride
